# Intraoperative mechanical ventilation practice in thoracic surgery patients and its association with postoperative pulmonary complications: results of a multicenter prospective observational study

**DOI:** 10.1186/s12871-020-01098-4

**Published:** 2020-07-22

**Authors:** Christopher Uhlig, Ary Serpa Neto, Meta van der Woude, Thomas Kiss, Jakob Wittenstein, Benjamin Shelley, Helen Scholes, Michael Hiesmayr, Marcos Francisco Vidal Melo, Daniele Sances, Nesil Coskunfirat, Paolo Pelosi, Marcus Schultz, Marcelo Gama de Abreu, LAS VEGAS# investigators, Protective Ventilation Network (PROVEnet), Clinical Trial Network of the European Society of Anaesthesiology

**Affiliations:** 1 Department of Anaesthesiology and Intensive Care Medicine, Pulmonary Engineering Group, University Hospital Carl Gustav Carus at the Technische Universität Dresden, Fetscherstr. 74, 01307 Dresden, Germany; 2 Department of Critical Care Medicine & Institute of Education and Research, Hospital Israelita Albert Einstein, São Paulo, Brazil; 3 Department of Intensive Care Medicine and Laboratory of Experimental Intensive Care and Anesthesiology, Academic Medical Center, University of Amsterdam, Amsterdam, The Netherlands; 4 Academic Unit of Anaesthesia, Pain and Critical Care, Golden Jubilee National Hospital / West of Scotland Heart and Lung Centre University of Glasgow, Glasgow, UK; 5 Division Cardiac, Thoracic, Vascular Anesthesia and Intensive Care, Medical University Vienna, Vienna, Austria; 6 Department of Anesthesia, Critical Care and Pain Medicine, Massachusetts General Hospital and Harvard Medical School, Boston, MA USA; 7 Division of Anaesthesiology and Intensive Care, IEO Istituto Europeo di Oncologia, Milan, Italy; 8 Department of Anaesthesiology and Reanimation, Akdeniz University Hospital, Antalya, Turkey; 9 Department of Surgical Sciences and Integrated Diagnostics, IRCCS San Martino IST, University of Genoa, Genoa, Italy

**Keywords:** Thoracic surgery, Mechanical ventilation, General anesthesia, Perioperative complications

## Abstract

**Background:**

Intraoperative mechanical ventilation may influence postoperative pulmonary complications (PPCs). Current practice during thoracic surgery is not well described.

**Methods:**

This is a post-hoc analysis of the prospective multicenter cross-sectional LAS VEGAS study focusing on patients who underwent thoracic surgery. Consecutive adult patients receiving invasive ventilation during general anesthesia were included in a one-week period in 2013. Baseline characteristics, intraoperative and postoperative data were registered. PPCs were collected as composite endpoint until the 5th postoperative day. Patients were stratified into groups based on the use of one lung ventilation (OLV) or two lung ventilation (TLV), endoscopic vs. non-endoscopic approach and ARISCAT score risk for PPCs. Differences between subgroups were compared using χ^2^ or Fisher exact tests or Student’s *t*-test. Kaplan–Meier estimates of the cumulative probability of development of PPC and hospital discharge were performed. Cox-proportional hazard models without adjustment for covariates were used to assess the effect of the subgroups on outcome.

**Results:**

From 10,520 patients enrolled in the LAS VEGAS study, 302 patients underwent thoracic procedures and were analyzed. There were no differences in patient characteristics between OLV vs. TLV, or endoscopic vs. open surgery. Patients received V_T_ of 7.4 ± 1.6 mL/kg, a PEEP of 3.5 ± 2.4 cmH_2_O, and driving pressure of 14.4 ± 4.6 cmH_2_O. Compared with TLV, patients receiving OLV had lower V_T_ and higher peak, plateau and driving pressures, higher PEEP and respiratory rate, and received more recruitment maneuvers. There was no difference in the incidence of PPCs in OLV vs. TLV or in endoscopic vs. open procedures. Patients at high risk had a higher incidence of PPCs compared with patients at low risk (48.1% vs. 28.9%; hazard ratio, 1.95; 95% CI 1.05–3.61; *p* = 0.033). There was no difference in the incidence of severe PPCs. The in-hospital length of stay (LOS) was longer in patients who developed PPCs. Patients undergoing OLV, endoscopic procedures and at low risk for PPC had shorter LOS.

**Conclusion:**

PPCs occurred frequently and prolonged hospital LOS following thoracic surgery. Proportionally large tidal volumes and high driving pressure were commonly used in this sub-population. However, large RCTs are needed to confirm these findings.

**Trial registration:**

This trial was prospectively registered at the Clinical Trial Register (www.clinicaltrials.gov; NCT01601223; registered May 17, 2012.)

## Background

Approximately 234 million major surgical procedures are undertaken worldwide every year [1]. Among these, approximately 7 million patients develop major complications resulting in one million deaths during surgery or in-hospital stay, contributing to an estimated mortality rate after anesthesia of 34 per million [1, 2]. According to the ‘Local assessment of ventilatory management during general anesthesia for surgery and effects on postoperative pulmonary complications’ (LAS VEGAS) trial, postoperative pulmonary complications (PPC) occur in a significant proportion of surgical patients [3]. However, since thoracic surgery requires a differentiated ventilatory approach, those patients were excluded from the primary analysis of the LAS VEGAS study. In thoracic surgery, conventional methods to prevent and treat hypoxemia during one lung ventilation (OLV) can be harmful to the lung tissue: high fraction of inspired oxygen (FIO2) and low (or no) positive end–expiratory pressure (PEEP) both can promote atelectasis, whereas high tidal volume (VT) can cause baro- and volutrauma [4]. The type of thoracic surgery (open or endoscopic) as well as the intraoperative mechanical ventilation settings may also influence PPCs.

Intraoperative mechanical ventilation with low V_T_, low driving pressure, and low to moderate PEEP improved postoperative lung function and even outcome in patients undergoing open abdominal surgery [5, 6]. When low V_T_ was used in abdominal surgery, high PEEP combined with recruitment maneuvers, as compared to low PEEP without recruitment maneuvers, did not add to the protection against PPCs [7].

The present study aimed to characterize the current mechanical ventilation practice during general anesthesia for thoracic surgery, describe the incidence of PPCs, and investigate possible associations between type of surgery (open vs. endoscopic), type of ventilation (OLV or two lung ventilation) and risk for PPCs (low risk vs high) with the incidence of PPCs. We hypothesized that intraoperative mechanical ventilation, as recommended in the literature, namely with low V_T_, low driving pressure, and low to moderate PEEP [8], is not commonly used during thoracic surgery, and that the incidence of PPCs is higher in this surgical population than in non-thoracic surgery.

## Methods

### Study design and sites

The present work is a post hoc analysis of the ‘Local assessment of ventilatory management during general anesthesia for surgery and effects on postoperative pulmonary complications’ (LAS VEGAS trial) [3]. The LAS VEGAS trial protocol was first approved by the institutional review board of the Academic Medical Center, Amsterdam, The Netherlands (W12_190#12.17.0227) and registered at clinicaltrials.gov (NCT01601223). The protocol of this trial was published elsewhere [9].

### Study population and data collection

Consecutive adult patients receiving invasive ventilation during general anesthesia for elective or non–elective surgery were eligible for participation in the study, which ran for seven predefined days in each country, selected by the national coordinator, in the period between January 14th and March 4th, 2013. Patients were excluded from participation if they were aged < 18 years, or scheduled for pregnancy related surgery, surgical procedures outside the operating room, or procedures involving cardio-pulmonary bypass.

The patient database of the LAS VEGAS trial was searched for eligible patients who received either open thoracic surgery, thoracoscopic or thoracoscopy assisted surgery (both summarized as endoscopic surgery), with or without OLV. These data have not been considered in previous analyses.

Reasonable parameters of baseline characteristics, intraoperative data and preoperative risk factors for PPCs were identified from previous studies [10–13]. During the intraoperative period, data describing ventilation settings and vital parameters, as well as episodes of hypoxia (SpO_2_ < 92%), use of recruitment maneuvers, airway pressure reduction, presence of expiratory flow limitation, hypotension (mean arterial pressure < 60 mmHg), use of vasoactive drugs, and new arrhythmias, was collected. Postoperative residual curarisation with neuromuscular blocking agents (NMBAs), defined as train–of–four stimulation (TOF) ratio <  0.9, was documented.

The definition of protective mechanical ventilation is still under debate. For this analysis it was based on recent recommendations [8, 14–16]. Patients were considered to be have been protectively ventilated “as recommended” if PEEP ≥5 cmH_2_O and V_T_ ≤ 8 ml/kg PBW during TLV [8, 14, 17], and PEEP ≥5 cmH_2_O and V_T_ ≤ 5 ml/kg PBW during OLV [18–20].

The occurrence of PPCs is presented as a collapsed composite of PPCs in the first five postoperative days. The following PPCs were scored daily from the day of surgery until hospital discharge or postoperative day 5: 1) need for supplementary oxygen (due to PaO_2_ < 60 mmHg or SpO_2_ < 90% in room air, excluding oxygen supplementation given as standard care or as continuation of preoperative therapy), 2) respiratory failure (PaO_2_ < 60 mmHg or SpO_2_ < 90% despite oxygen therapy, or need for non-invasive mechanical ventilation), 3) unplanned new or prolonged invasive *or* non–invasive mechanical ventilation, 4) acute respiratory distress syndrome, 5) pneumonia. Severe PPCs were defined as the occurrence of one or more of the complications 2–5. Patient data were anonymized before entry onto a password secured, web–based electronic case record form (OpenClinica, Boston, MA, USA).

### Statistical analysis

Patients were stratified into groups based on: 1) use or not of OLV (OLV vs. only TLV); 2) use or not of an endoscopic approach (endoscopic vs. open); and 3) risk for PPC according to ARISCAT (low risk [ARISCAT < 26] vs. moderate-to-high risk [ARISCAT ≥26] Supplemental Table 2, Additional file 1). The ventilatory data, which were collected hourly, were first averaged for each patient according to the number of observations (median of the value). In a longitudinal analysis, this data is presented for the first, second, third, fourth and last hour of surgery. All data are presented for the whole population and for the subgroups. In-hospital length of stay (LOS) and in-hospital mortality was censored at postoperative day 28. Proportions are compared using χ^2^ or Fisher exact tests and continuous variables are compared using the Mann-Whitney *U* Test, as appropriate.

The distributions of combinations of tidal volume size and PEEP level are presented in scatter plots. Cut-offs of 6 ml/kg PBW for tidal volume, and 5 cmH_2_O for PEEP were chosen to form the matrices. These cut-offs were based on widely accepted values of each variable, or according to normal daily practice. The driving pressure was defined as plateau pressure (Pplat) minus the PEEP level.

Kaplan–Meier estimates of the cumulative probability of development of PPC and hospital discharge were performed. Cox proportional hazard models without adjustment for covariates were used to assess the effect of the subgroups on outcome. The proportionality assumption was tested with scaled Schoenfeld residuals. Adjustments for multiple comparisons were not performed and no assumption for missing data was done. Statistical significance was considered to be at two-sided *p* <  0.05. All analyses were performed with R version 3.4.1 (http://www.R-project.org/).

## Results

From 10,520 patients enrolled in the LAS VEGAS study, 302 patients underwent thoracic procedures (Supplemental Figure 1, Additional file 1). Characteristics of patient and surgery are shown in Table 1. In this sub-population of 302 thoracic surgical patients, 55% (168/302) received OLV, 15.2% (46/302) were operated with an endoscopic approach and 87.4% (264/302) had moderate-to-high risk for PPCs.

**Table 1:** Pre-Operative Characteristics of the Patients According to Subgroups

	All Patients (***n*** = 302)	OLV (***n*** = 168)	TLV (***n*** = 134)	***p*** value	Endoscopic (***n*** = 46)	Open (***n*** = 256)	***p*** value	Low Risk (***n*** = 38)	High Risk (***n*** = 264)	***p*** value
Age, years	62.0 (50.0–70.8)	62.0 (50.0–70.0)	62.0 (47.2–71.0)	0.799	62.0 (49.8–70.8)	62.0 (50.0–70.2)	0.839	37.0 (28.2–46.0)	64.0 (54.8–71.2)	< 0.001
Male gender	181 (59.9)	101 (60.1)	80 (59.7)	0.941	23 (50.0)	158 (61.7)	0.135	17 (44.7)	164 (62.1)	0.040
BMI, kg/m^2^	25.6 (22.9–28.9)	25.7 (23.3–29.3)	25.4 (22.5–28.7)	0.398	25.1 (21.8–27.5)	25.7 (23.0–29.3)	0.129	23.1 (20.9–26.3)	25.8 (23.4–29.2)	0.010
ASA physical status				0.573			0.880			< 0.001
1	40 (13.3)	22 (13.1)	18 (13.5)		5 (11.1)	35 (13.7)		20 (54.1)	20 (7.6)	
2	109 (36.2)	60 (35.7)	49 (36.8)		18 (40.0)	91 (35.5)		17 (45.9)	92 (34.8)	
3	141 (46.8)	82 (48.8)	59 (44.4)		20 (44.4)	121 (47.3)		0 (0.0)	141 (53.4)	
4	11 (3.7)	4 (2.4)	7 (5.3)		2 (4.4)	9 (3.5)		0 (0.0)	11 (4.2)	
ARISCAT score	40.0 (27.0–49.5)	40.0 (27.0–47.0)	39.0 (27.0–50.0)	0.755	35.0 (27.0–43.0)	40.0 (27.0–50.0)	0.018	24.0 (24.0–24.0)	43.0 (27.0–50.0)	< 0.001
Functional status										
Independent	270 (89.4)	153 (91.1)	117 (88.0)	0.536	43 (93.5)	227 (89.0)	0.438	36 (94.7)	234 (89.0)	
Partially dependent	26 (8.6)	12 (7.1)	14 (10.5)		2 (4.3)	24 (9.4)		2 (5.3)	24 (9.1)	0.772
Totally dependent	5 (1.7)	3 (1.8)	2 (1.5)		1 (2.2)	4 (1.6)		0 (0.0)	5 (1.9)	
Smoking	83 (27.5)	43 (25.6)	40 (29.9)	0.410	12 (26.1)	71 (27.7)	0.817	9 (23.7)	74 (28.0)	0.574
Transfusion (< 24 h)	6 (2.0)	2 (1.2)	4 (3.0)	0.411	0 (0.0)	6 (2.3)	0.595	0 (0.0)	6 (2.3)	1.000
Respiratory infection (< 30 d)	24 (7.9)	17 (10.1)	7 (5.2)	0.118	2 (4.3)	22 (8.6)	0.551	0 (0.0)	24 (9.1)	0.054
Recent MV (< 30 d)	8 (2.6)	5 (3.0)	3 (2.2)	1.000	0 (0.0)	8 (3.1)	0.612	0 (0.0)	8 (3.0)	0.602
Co-morbidities										
COPD	54 (17.9)	35 (20.8)	19 (14.2)	0.133	6 (13.0)	48 (18.8)	0.352	1 (2.6)	53 (20.1)	0.005
Apnea	5 (1.7)	1 (0.6)	4 (3.0)	0.174	1 (2.2)	4 (1.6)	0.564	0 (0.0)	5 (1.9)	1.000
Liver cirrhosis	2 (0.7)	2 (1.2)	0 (0.0)	0.504	0 (0.0)	2 (0.8)	1.000	0 (0.0)	2 (0.8)	1.000
Chronic kidney failure	7 (2.3)	5 (3.0)	2 (2.0)	0.468	1 (2.2)	6 (2.3)	1.000	0 (0.0)	7 (2.7)	0.601
Heart failure	23 (7.6)	9 (5.4)	14 (10.4)	0.097	2 (4.3)	21 (8.2)	0.548	0 (0.0)	23 (8.7)	0.092
Neuro disease	8 (2.6)	5 (5.0)	3 (2.2)	1.000	2 (4.3)	6 (2.3)	0.350	2 (5.3)	6 (2.3)	0.265
**Laboratorial tests and vital signs**										
SpO_2_, %	97.0 (95.0–98.0)	97.0 (95.0–98.0)	97.0 (95.0–98.0)	0.347	98.0 (95.0–99.0)	97.0 (95.0–98.0)	0.232	98.0 (97.0–99.0)	97.0 (95.0–98.0)	< 0.001
Hemoglobin, g/dL	13.5 (12.4–14.8)	13.5 (12.4–14.5)	13.4 (12.5–15.0)	0.700	13.3 (12.9–14.6)	13.6 (12.3–14.8)	0.773	14.3 (13.3–15.2)	13.4 (12.3–14.6)	0.002
WBC, cell/mm^3^	7150.0 (6000.0–9000.0)	7000.0 (6000.0–9000.0)	7565.0 (6000.0–9150.0)	0.755	8000.0 (6000.0–9912.5)	7000.0 (6000.0–9000.0)	0.344	7000.0 (6000.0–8125.0)	7300.0 (6000.0–9000.0)	0.506
**Surgical characteristics**										
Procedure^a^										
Vascular	2 (0.7)	0 (0.0)	2 (1.5)	0.196	0 (0.0)	2 (0.8)	1.000	0 (0.0)	2 (0.8)	1.000
Cardiac	12 (4.0)	0 (0.0)	12 (9.0)	< 0.001	1 (2.2)	11 (4.3)	0.700	0 (0.0)	12 (4.5)	0.374
Lung / Pleural	231 (76.5)	152 (90.5)	79 (59.0)	< 0.001	34 (73.9)	197 (77.0)	0.654	27 (71.1)	204 (77.3)	0.397
Other	64 (21.2)	21 (12.5)	43 (32.1)	< 0.001	11 (23.9)	53 (20.7)	0.623	12 (31.6)	52 (19.7)	0.093
Condition										
Elective	283 (93.7)	160 (95.2)	123 (91.8)	0.454	41 (91.3)	241 (94.1)	0.148	36 (94.7)	247 (93.6)	
Urgency	13 (4.3)	5 (3.0)	8 (6.0)		4 (8.7)	9 (3.5)		2 (5.3)	11 (4.2)	0.856
Emergency	6 (2.0)	3 (1.8)	3 (2.2)		0 (0.0)	6 (2.3)		0 (0.0)	6 (2.3)	
Planned duration										
≤ 2 h	136 (45.0)	73 (43.5)	63 (47.0)	0.427	28 (60.9)	108 (42.2)	0.061	30 (78.9)	106 (40.2)	
2–3 h	95 (31.5)	58 (34.5)	37 (27.6)		11 (23.9)	84 (32.8)		6 (15.8)	89 (33.7)	< 0.001
> 3 h	71 (23.5)	37 (22.0)	34 (25.4)		7 (15.2)	64 (25.0)		2 (5.3)	69 (26.1)	
Antibiotic prophylaxis	266 (88.1)	146 (86.9)	120 (89.6)	0.480	38 (82.6)	228 (89.1)	0.213	27 (71.1)	239 (90.5)	< 0.001
Epidural anesthesia	72 (23.8)	48 (28.6)	24 (17.9)	0.307	5 (10.9)	67 (26.2)	0.024	4 (10.5)	68 (25.8)	0.039
Type of tube										
Endotracheal	84 (27.8)	11 (6.5)	73 (54.5)	< 0.001	24 (52.2)	60 (23.4)	0.001	15 (39.5)	69 (26.1)	
Bronchus blocker	19 (6.3)	12 (7.1)	7 (5.2)		1 (2.2)	18 (7.0)		2 (5.3)	17 (6.4)	0.295
SGA	6 (2.0)	2 (1.2)	4 (3.0)		0 (0.0)	6 (2.3)		1 (2.6)	5 (1.9)	
DLT	193 (63.9)	143 (85.1)	50 (37.3)		21 (45.7)	172 (67.2)		20 (52.6)	173 (65.5)	
Duration of surgery, min	105.0 (55.0–174.2)	104.0 (57.5–164.8)	105.0 (55.0–180.0)	0.836	62.5 (45.0–131.2)	110.0 (62.2–180.0)	0.003	55.0 (41.2–80.0)	115.0 (62.2–180.8)	< 0.001
Duration of anesthesia, min	145.0 (90.0–225.0)	147.5 (97.8–225.0)	145.0 (90.0–235.0)	0.651	105.0 (80.0–153.8)	152.0 (100.0–236.0)	0.001	88.0 (64.5–120.0)	160.0 (100.0–240.0)	< 0.001

Values are presented as median (interquartile range) or number (percentage). *p* values from a Proportions χ2 or Fisher exact tests for proportions and Mann-Whitney *U* Test for continuous variables

*ARISCAT: ASA* American Society of Anesthesiology recommended physical status, *BMI* Body mass index*, COPD* Chronic obstructive pulmonary disease, *DLT* Double-lumen tube, *MV* Mechanical ventilation, *OLV* One-lung ventilation*, SGA* Supraglottic airway, *SpO*_*2*_ Pulse oximetry, *TLV* Total lung ventilation, *WBC* White blood count;

^a^more than one option allowed

Characteristics of patients undergoing procedures with OLV vs. TLV, and endoscopic vs. open were comparable. Patients with moderate-to-high risk for PPCs were different from those at low risk with respect to age, gender, BMI, ASA status, COPD prevalence and planned duration of surgery (Table 1).

### Intra-operative characteristics

Patients operated under OLV received more often double-lumen tubes and had more frequently lung or pleural surgery than those operated under TLV (Table 1). Use of epidural anesthesia was less and duration of surgery shorter in endoscopic compared to non-endoscopic surgery (Table 1).

Patients at moderate-to-high risk for PPC received more frequently antibiotic prophylaxis and epidural anesthesia, and had longer duration of surgery as well as anesthesia, compared with patients at low risk (Table 1).

The amounts of crystalloids, colloids, albumin and packed red blood cells was higher in open vs. endoscopic surgery, and in patients at moderate-to-high vs. low risk for PPC (Table 2).

**Table 2:** Intra-Operative Characteristics of the Patients According to Subgroups

	All Patients (***n*** = 302)	OLV (***n*** = 168)	TLV (***n*** = 134)	***p*** value	Endoscopic (***n*** = 46)	Open (***n*** = 256)	***p*** value	Low Risk (***n*** = 38)	High Risk (***n*** = 264)	***p*** value
**Ventilation and vital signs**										
Ventilatory mode										
Volume controlled	209 (70.8)	121 (73.3)	88 (67.7)	0.055	28 (65.1)	181 (71.8)	0.285	32 (91.4)	177 (68.1)	
Pressure controlled	51 (17.3)	28 (17.0)	23 (17.7)		7 (16.3)	44 (17.5)		2 (5.7)	49 (18.8)	
Pressure support	3 (1.2)	3 (1.8)	0 (0.0)		0 (0.0)	3 (1.2)		0 (0.0)	3 (1.2)	0.109
Spontaneous	8 (2.7)	1 (0.6)	7 (5.4)		3 (7.0)	5 (2.0)		0 (0.0)	8 (3.1)	
Other	24 (8.1)	12 (7.3)	12 (9.2)		5 (11.6)	19 (7.5)		1 (2.9)	23 (8.8)	
V_T_, ml	472.2 (400.5–525.0)	453.5 (398.4–510.0)	475.0 (430.6–549.0)	0.015	468.8 (400.0–544.5)	472.2 (405.6–525.0)	0.895	483.2 (443.1–543.9)	468.5 (400.0–525.0)	0.102
V_T_, ml/kg PBW^a^	7.6 (6.3–8.4)	7.4 (6.0–8.3)	7.6 (6.6–8.5)	0.050	7.6 (6.2–8.8)	7.5 (6.3–8.3)	0.225	7.6 (7.0–8.3)	7.5 (6.2–8.4)	0.587
Peak pressure, cmH_2_O^a^	20.0 (17.5–24.0)	21.0 (18.0–25.0)	19.0 (16.0–23.0)	0.001	20.0 (16.2–23.0)	20.0 (18.0–24.0)	0.187	18.5 (15.6–22.0)	20.0 (18.0–24.1)	0.068
Plato pressure, cmH_2_O^a^	17.8 (15.0–21.0)	18.2 (16.0–21.4)	16.5 (13.1–20.0)	0.004	16.0 (12.5–20.2)	18.0 (15.0–21.0)	0.118	15.8 (13.0–18.9)	18.0 (15.5–21.0)	0.010
PEEP, cmH_2_O^a^	4.0 (1.5–5.0)	4.5 (2.4–5.0)	3.0 (1.5–5.0)	0.006	3.0 (1.5–5.0)	4.0 (2.0–5.0)	0.176	2.0 (0.0–5.0)	4.0 (2.0–5.0)	0.008
Driving pressure, cmH_2_O^a^	14.0 (11.0–17.0)	14.5 (12.0–17.5)	13.0 (10.5–16.0)	0.016	13.0 (10.8–16.0)	14.0 (11.5–17.0)	0.389	14.0 (10.5–16.0)	14.0 (11.5–17.0)	0.216
Respiratory rate, bpm	12.0 (12.0–14.0)	12.0 (12.0–15.0)	12.0 (12.0–14.0)	0.128	12.0 (11.1–14.4)	12.0 (12.0–14.0)	0.715	12.0 (12.0–13.5)	12.0 (12.0–14.5)	0.644
FiO_2_, %	65.0 (50.0–80.0)	65.8 (50.0–85.0)	63.5 (50.0–80.0)	0.294	70.0 (58.5–83.6)	64.5 (50.0–80.0)	0.151	68.2 (50.0–75.0)	65.0 (50.0–84.0)	0.543
SpO_2_, %	99.0 (97.5–100.0)	98.5 (97.0–100.0)	99.0 (98.0–100.0)	0.039	99.0 (98.0–99.9)	99.0 (97.5–100.0)	0.724	99.0 (98.0–100.0)	99.0 (97.5–100.0)	0.352
MAP, mmHg	78.0 (71.0–86.6)	78.0 (71.0–86.0)	78.0 (71.0–88.0)	0.940	80.8 (74.0–95.0)	77.5 (70.0–85.0)	0.009	77.5 (71.5–91.6)	78.0 (71.0–86.0)	0.474
Heart rate, bpm	73.5 (64.5–82.0)	73.5 (64.5–82.5)	73.5 (65.0–81.1)	0.893	73.2 (65.2–79.0)	74.0 (64.5–82.0)	0.663	75.0 (70.0–81.5)	73.5 (63.6–82.0)	0.223
RM	105 (34.8)	71 (42.3)	34 (25.4)	0.002	13 (28.3)	92 (35.9)	0.314	7 (18.4)	98 (37.1)	0.023
In the last hour	68 (22.5)	52 (31.0)	16 (11.9)	< 0.001	6 (13.0)	62 (24.2)	0.094	2 (5.3)	66 (25.0)	0.006
Number of RM	0.0 (0.0–1.0)	0.0 (0.0–1.0)	0.0 (0.0–0.8)	0.003	0.0 (0.0–1.0)	0.0 (0.0–1.0)	0.397	0.0 (0.0–0.0)	0.0 (0.0–1.0)	0.020
Protective ventilation	41 (14.8)	13 (8.3)	28 (23.5)	< 0.001	10 (24.4)	31 (13.2)	0.091	4 (11.4)	37 (15.4)	0.798
**Anesthesia characteristics**										
Type of anesthesia										
TIVA	56 (18.5)	25 (14.9)	31 (23.1)	0.012	10 (21.7)	46 (18.0)	0.287	6 (15.8)	50 (18.9)	
Volatile	188 (62.3)	117 (69.6)	71 (53.0)		31 (67.4)	157 (61.3)		22 (57.9)	166 (62.9)	0.483
Mixed	58 (19.2)	26 (15.5)	32 (23.9)		5 (10.9)	53 (20.7)		10 (26.3)	48 (18.2)	
Opioids										
Short acting	66 (21.9)	39 (23.2)	27 (20.3)	0.151	14 (30.4)	52 (20.4)	0.306	11 (28.9)	55 (20.9)	
Long acting	202 (66.9)	106 (63.1)	96 (72.2)		28 (60.9)	174 (68.2)		24 (63.2)	178 (67.7)	0.475
Both	33 (10.9)	23 (13.7)	10 (7.5)		4 (8.7)	29 (11.4)		3 (7.9)	30 (11.4)	
Total Fluids										
Crystalloids, ml	1000.0 (875.0–2000.0)	1000.0 (1000.0–1750.0)	1000.0 (800.0–2000.0)	0.949	900.0 (500.0–1100.0)	1130.0 (1000.0–2000.0)	< 0.001	1000.0 (670.0–1000.0)	1100.0 (1000.0–2000.0)	< 0.001
Colloids, ml	500.0 (67.5–700.0)	500.0 (0.0–500.0)	500.0 (500.0–1000.0)	0.076	0.0 (0.0–500.0)	500.0 (450.0–850.0)	0.027	0.0 (0.0–500.0)	500.0 (500.0–1000.0)	0.007
Albumin, ml	0.0 (0.0–0.0)	0.0 (0.0–0.0)	0.0 (0.0–12.2)	0.755	0.0 (0.0–0.0)	0.0 (0.0–0.0)	0.212	0.0 (0.0–0.0)	0.0 (0.0–0.0)	0.134
PRBC, units	0.0 (0.0–2.0)	0.0 (0.0–1.0)	1.0 (0.0–2.0)	0.162	0.0 (0.0–0.0)	0.0 (0.0–2.0)	0.045	0.0 (0.0–0.0)	0.0 (0.0–2.0)	0.017
Reversal of NMBA	115 (38.1)	72 (43.1)	43 (32.1)	0.050	16 (34.8)	99 (38.8)	0.603	14 (36.8)	101 (38.4)	0.853

Values are presented as median (interquartile range) or number (percentage). *p* values from a Proportions χ2 or Fisher exact tests for proportions and Mann-Whitney *U* Test for continuous variables *bpm* beats per minute, *etCO*_*2*_ End-tidal carbon dioxide, *FiO*_*2*_ Inspired fraction of oxygen, *MAP* Mean arterial pressure, *mpm* Movements per minute, *NMBA* Neuromuscular blocking agents, *OLV* One lung ventilation, *PBW* Predicted body weight, *PEEP* Positive end-expiratory pressure, *PRBC* Packed red blood cells, *RM* Recruitment maneuver, *SpO*_*2*_ Pulse oximetry, *TIVA* Total intravenous anesthesia, *TLV* Total lung ventilation, *V*_*T*_ Tidal volume

^a^data presented as the median used through surgery

### Mechanical ventilation

Patients were ventilated with V_T_ of 7.4 ± 1.6 ml/kg PBW, PEEP of 3.5 ± 2.4 cmH_2_O, and driving pressure of 14.4 ± 4.6 cmH_2_O (Table 2). Compared to patients operated solely under TLV, patients receiving OLV had lower V_T_, higher peak, plateau and driving pressures, as well as PEEP and respiratory rate, and received higher number of recruitment maneuvers (Table 2). Protective ventilation was used in 14.8% (41/302) of all patients, mainly during TLV. The ventilatory management of patients undergoing endoscopic and non-endoscopic procedures did not differ significantly. Patients at moderate-to-high risk for PPC had higher levels of PEEP, and received more recruitment maneuvers than patients at low risk (Table 2).

Values of ventilator settings along time are shown in Supplemental Figures 2 through 4 (Additional file 1). Patients operated under OLV had higher FiO_2_ compared with patients operated under TLV (Supplemental Figure 2, Additional file 1). The combinations of V_T_ and PEEP according to subgroups are shown in Supplemental Figures 5 through 7 (Additional file 1).

### Primary outcome

**Fig. 1:**
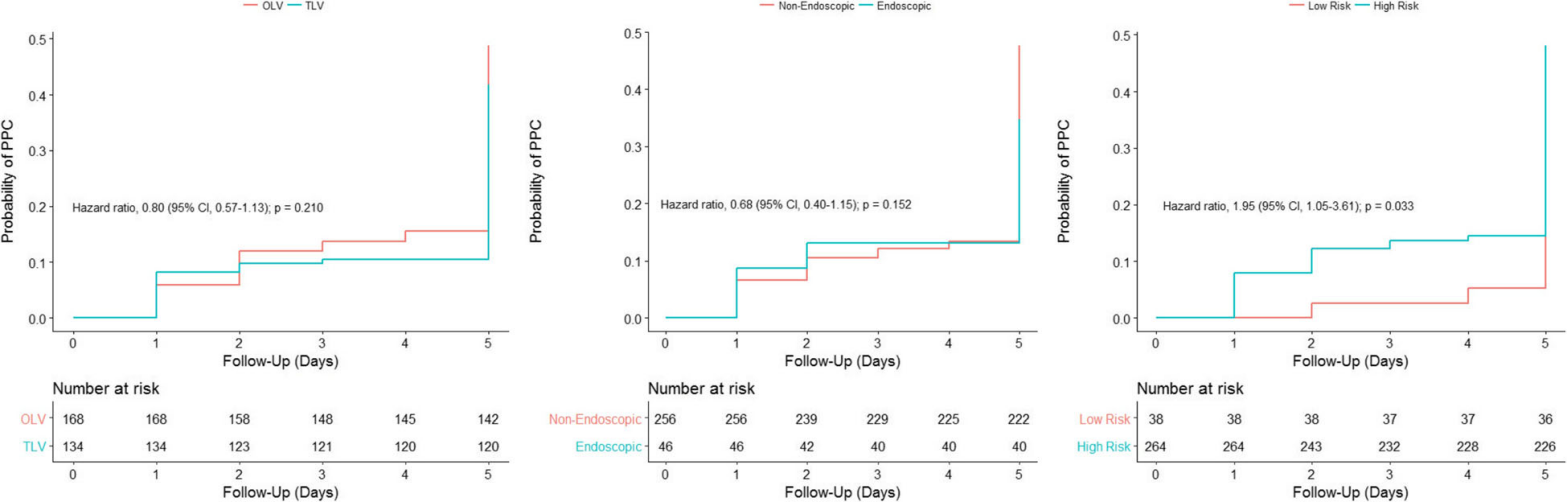
Probability of PPC according to the subgroups assessed. *PPC: postoperative pulmonary complications; OLV: one-lung ventilation; TLV: two-lung ventilation* Non-adjusted hazard ratios.

The overall incidence of PPCs in this population was 45.7% (138/302), and did not differ significantly between OLV vs. TLV (82/168 vs. 56/134, 48.8% vs. 41.8%, *p* = 0.223, total number and percentage respectively), and endoscopic vs. open procedures (16/46 vs. 122/256, 34.8% vs. 47.7%, *p* = 0.106, total number and percentage respectively, Table 3, Fig. 1). Patients at moderate-to-high risk showed an increased incidence of PPC compared to patients at lower risk (48.1% vs. 28.9%; hazard ratio, 1.95; 95% CI 1.05–3.61; *p* = 0.033), mainly due to unplanned need for supplemental oxygen (Table 3, Fig. 1).

**Table 3:** Clinical Outcomes of the Patients According to Subgroups

	All Patients (***n*** = 302)	OLV (***n*** = 168)	TLV (***n*** = 134)	***p*** value	Endoscopic (***n*** = 46)	Open (***n*** = 256)	***p*** value	Low Risk (***n*** = 38)	High Risk (***n*** = 264)	***p*** value
**Primary outcome**										
PPC	138 (45.7)	82 (48.8)	56 (41.8)	0.223	16 (34.8)	122 (47.7)	0.106	11 (28.9)	127 (48.1)	0.026
Need of oxygen	109 (36.1)	65 (38.9)	44 (32.8)	0.274	12 (26.1)	97 (38.0)	0.120	8 (21.1)	101 (38.4)	0.037
Respiratory failure	26 (8.6)	15 (8.9)	11 (8.2)	0.824	1 (2.2)	25 (9.8)	0.148	2 (5.3)	24 (9.1)	0.755
Invasive MV	26 (8.6)	14 (8.3)	12 (9.0)	0.848	3 (6.5)	23 (9.0)	0.778	1 (2.6)	25 (9.5)	0.222
NIV	15 (5.0)	10 (6.0)	5 (3.7)	0.377	2 (4.3)	13 (5.1)	1.000	1 (2.6)	14 (5.3)	0.702
ARDS	4 (1.3)	4 (2.4)	0 (0.0)	0.132	0 (0.0)	4 (1.6)	1.000	0 (0.0)	4 (1.5)	1.000
Pneumonia	8 (2.6)	6 (3.6)	2 (1.5)	0.307	2 (4.3)	6 (2.3)	0.350	1 (2.6)	7 (2.7)	1.000
**Secondary outcomes**										
Severe PPC^a^	53 (17.5)	30 (17.9)	23 (17.2)	0.875	7 (15.2)	46 (18.0)	0.651	4 (10.5)	49 (18.6)	0.223
Intra-OP complications										
Desaturation	61 (20.2)	38 (22.6)	23 (17.2)	0.240	7 (15.2)	54 (21.1)	0.360	5 (13.2)	56 (21.2)	0.247
Unplanned RM	48 (15.9)	31 (18.5)	17 (12.7)	0.173	7 (15.2)	41 (16.0)	0.891	3 (7.9)	45 (17.0)	0.149
Pressure reduction	36 (11.9)	27 (16.1)	9 (6.7)	0.012	5 (10.9)	31 (12.1)	0.811	2 (5.3)	34 (12.9)	0.281
Flow limitation	3 (1.0)	2 (1.2)	1 (0.8)	1.000	0 (0.0)	3 (1.2)	1.000	0 (0.0)	3 (1.1)	1.000
Hypotension	102 (33.8)	60 (35.7)	42 (31.3)	0.424	8 (17.4)	94 (36.7)	0.010	3 (7.9)	99 (37.5)	< 0.001
Vasopressors	113 (37.4)	65 (38.7)	48 (35.8)	0.608	13 (28.3)	100 (39.1)	0.163	3 (7.9)	110 (41.7)	< 0.001
New arrhythmias	6 (2.0)	3 (1.8)	3 (2.2)	1.000	0 (0.0)	6 (2.3)	0.595	0 (0.0)	6 (2.3)	1.000
ICU admission^b^	6 (2.0)	2 (1.2)	4 (3.0)	0.411	0 (0.0)	6 (2.3)	0.595	0 (0.0)	6 (2.3)	1.000
Hospital LOS, days	6.0 (3.0–10.0)	6.0 (4.0–11.0)	5.0 (3.0–9.0)	0.010^**c**^	3.0 (1.0–7.5)	6.0 (4.0–10.0)	< 0.001^**c**^	4.0 (1.0–6.0)	6.0 (4.0–10.0)	< 0.001^**c**^
Hospital mortality	1 (0.3)	1 (0.6)	0 (0.0)	1.000	0 (0.0)	1 (0.4)	1.000	0 (0.0)	1 (0.4)	1.000

Values are presented as median (interquartile range) or number (percentage). *p* values from a Proportions χ2 or Fisher exact tests for proportions and Mann-Whitney *U* Test for continuous variables *ARDS* Acute respiratory distress syndrome, *ICU* Intensive care unit, *Intra-OP* Intraoperative, *LOS* Length of stay, *MV* Mechanical ventilation, *NIV* Non-invasive ventilation, *OLV* One lung ventilation, *PPC* Postoperative pulmonary complication, *RM* Recruitment maneuvers, *TLV* Total lung ventilation

^a^excluding need of oxygen

^b^unplanned admission

^c^*p* value from the Cox proportional hazard model

### Secondary outcomes

The incidence of severe PPCs, unplanned ICU admission and hospital mortality did not differ among groups (Table 3). The incidence of hypotension was decreased in endoscopic compared to open procedures, and in patients at lower compared to moderate-to-high risk of PPCs (Table 3).

**Fig. 2:**
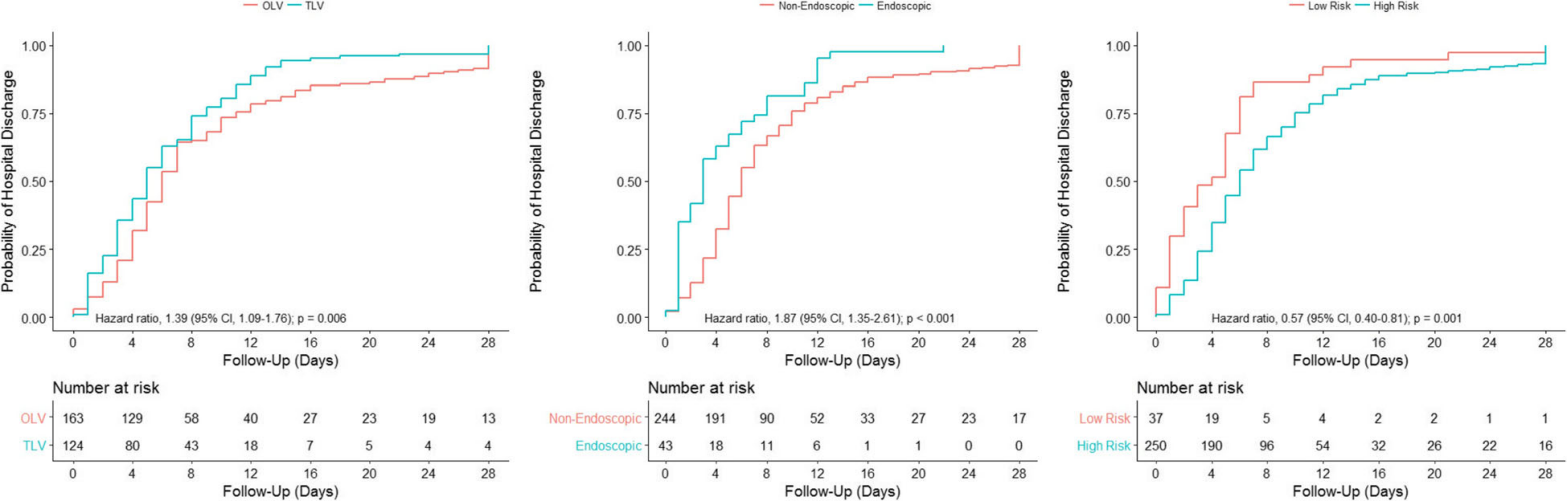
Probability of hospital discharge according to the subgroups assessed. *OLV: one-lung ventilation; TLV: two-lung ventilation*. Non-adjusted hazard ratios

The LOS was increased in patients who developed PPCs (Supplemental Figure 8, Additional file 1), and shorter in patients operated under OLV vs. TLV, endoscopic vs. open, and those with low vs. moderate-to-high risk for PPC (Table 3, Fig. 2).

## Discussion

In this population of patients undergoing thoracic surgery: 1) mechanical ventilation differed from those recommended for lung protection in 85.2% of all patients; 2) patients under OLV received lower V_T_, higher peak, plateau and driving pressures, higher PEEP levels and respiratory rate, and received more recruitment maneuvers compared with TLV; 3) the overall incidence of PPCs was as high as 45.7%; 4) PPCs were more common among patients with higher ARISCAT score or co-morbidities, but not increased following open vs. endoscopic procedures, or OLV vs. TLV; 6) PPCs were associated with increased LOS.

To our knowledge, this is the first prospective observational investigation addressing the practice of mechanical ventilation and incidence of PPCs in thoracic anesthesia. The main strengths of our study are that data was stored, analyzed and reported according to international standards [21].

High V_T_ strategies, usually accompanied by low or zero PEEP, have been used to prevent intraoperative atelectasis [22, 23]. However, this may cause overdistension (volutrauma), and repetitive collapse-reopening of lung units (atelectrauma), which can injure the lungs and lead to PPCs [24]. A protective ventilation approach consisting mainly of low V_T_ reduces the incidence of PPCs [7, 25]. This seems to apply also to thoracic anesthesia but this claim is not undisputed [26–28]. The present study shows that protective mechanical ventilation, as recommended, was used in less than 15% of patients undergoing thoracic surgery. Different possible reasons might explain this finding: 1) the concept of protective ventilation during surgery is still not widespread among anesthesiologists; 2) the role of single components of mechanical ventilation in lung protection, especially of PEEP, is still poorly defined, leading anesthesiologists to set values according to their own preferences; 3) sound evidence from large RCTs demonstrating the benefit of protective mechanical ventilation in thoracic surgical patients is still missing; 4) thoracic surgical procedures usually last less than 1 hour, which might be deemed as too short to benefit from protective mechanical ventilation; 5) mechanical ventilation settings guided by driving pressure may result in V_T_ and PEEP outside the range that has been recommended for protective mechanical ventilation.

The incidence of PPCs after surgery is influenced by patient-related factors, and type of surgery. In a mixed surgical population without surgery involving cardiopulmonary bypass, 10.4% of patients developed PPCs within the first postoperative 5 days; values ranged from 6.7% in plastic/cutaneous procedures to 38.2% in transplant surgery [3]. In open abdominal surgery, PPCs were reported in 10.5 to 39.0% of patients, despite the use of a protective ventilation strategy [3, 7, 25]. In average, 10.7% of patients at increased risk, for example obese patients, developed PPCs [29]. In patients undergoing thoracic surgery, an incidence of PPCs between 10.7 and 50% has been reported [26, 30–32]. This relatively wide range is possibly explained by differences in definition of pulmonary complications among trials. The rate of severe PPCs was 17.5% in our thoracic surgery population, which is comparable to the rate of 18.1% reported by Blank and colleagues [26].

The observation that patients who developed PPCs had more comorbidities and longer LOS is in line with previous studies addressing intraoperative TLV [3, 33]. The difference in LOS in the subgroups is likely explained by the type of procedure per se, where open approaches require a prolonged treatment due to more complex procedures, independent from the type of mechanical ventilation.

Although the incidence of PPCs was relatively high, neither open thoracic surgery procedures, nor OLV itself were associated with them, especially when taking the infrequent use of protective mechanical ventilation in this population into account. The precise role of PEEP for protective intraoperative mechanical ventilation has been challenged in recent trials [7, 34]. In fact, it has been suggested that a strategy aimed at permissive atelectasis might be as protective as a strategy to open lungs during surgery [14, 35]. Our finding that higher V_T_ was not associated with PPCs is intriguingly, but in agreement with data from an observational study reporting that the use of V_T_ as high as 8 mL/kg as even associated with better pulmonary outcome [26]. Together, these findings suggest that protective OLV settings are more complex than previously thought. Cutoff values, although valuable, must not only consider the interaction among variables, but also a possible role of airway pressures.

### Limitations

This study has several limitations. First, a one-week inclusion period was relatively short in order to include a high number of patients per center. However, this fact was counterbalanced by the multicenter design. Second, a short inclusion period might have resulted in selection bias, since fluctuation of the severity of cases cannot be ruled out. Nevertheless, the benefits of avoiding changes in therapy during the observation period as a potential confounder should not be underestimated. Third, the definition of protective mechanical ventilation was based on recommendations that are still under debate. Fourth, most study sites included less than 10 patients. This number, however, does not imply lack of experience with the procedure, since thoracic anesthesia per se already requires a substantial degree of expertise. Fifth, the duration of OLV was not investigated and, therefore, the exact contribution of OLV to PPCs cannot be separated from the period under TLV in this sub-population. Sixth, the design of this study precludes the possibility of determining cause-effect relationships, and results must be seen from a hypothesis-generating perspective. Seventh, the fact that data was collected prospectively might have interfered with clinical practice itself, and biased towards the use of protective ventilation. Still, non-protective ventilation was used in a vast majority of patients. Eighth, the total number of patients enrolled allowed analyses of three subgroups only. Potential confounders could be the type of anesthesia (total intravenous anesthesia vs. volatile anesthetics), the type of postoperative analgesia (epidural anesthesia vs. opioids) or the ASA status, which should be subject of future trials.

## Conclusions

The present study provides relevant insight into the practice of mechanical ventilation during thoracic surgery. The data might prove useful for the development of scores for risk prediction in this particular population, allocation of human and financial resources, including need for postoperative monitoring in dedicated units, and also estimation of sample size in interventional trials [18]. Mechanical ventilation practice did not follow current recommendations for lung protection in the vast majority of patients undergoing thoracic surgery. Although PPCs were common in this population, and associated with increased LOS, their incidence was not higher following open vs. endoscopic or OLV vs. TLV, and not associated with mechanical ventilation settings. It must be emphasized that the lack of association between mechanical ventilation settings and PPCs does not support use of non-protective V_T_ and PEEP in this population.

## Supplementary information

**Additional file 1:** This PDF file contains a list of the LAS VEGAS Thorax study collaborators; **Table S1.** Participating centers; **Table S2.** ARISCAT Risk Score; **Figure S1.** Flowchart; **Figure S2.** Tidal volume, driving pressure, PEEP and FiO_2_ over time according to the use of one-lung ventilation or two-lung ventilation; **Figure S3.** Tidal volume, driving pressure, PEEP and FiO_2_ over time in endoscopic or open procedures; **Figure S4.** Tidal volume, driving pressure, PEEP and FiO_2_ over time according to the risk for PPC; **Figure S5.** Combinations of tidal volume and PEEP in the first three hours and last hour of surgery according to the use of one-lung ventilation or two-lung ventilation; **Figure S6.** Combinations of tidal volume and PEEP in the first three hours and last hour of surgery in endoscopic or non-endoscopic procedures; **Figure S7.** Combinations of tidal volume and PEEP in the first three hours and last hour of surgery according to the risk for PPC and **Figure S8.** Probability of hospital discharge according to development of PPC.

## Data Availability

The datasets used and/or analyzed during the current study are available from the corresponding author on reasonable request.

## Abbreviations

ARISCAT Score: Assess Respiratory Risk in Surgical Patients in Catalonia Score
ESA: European Society of Anaesthesiology
F_I_O_2_: Fraction of inspired oxygen
LAS VEGAS: Local assessment of ventilatory management during general anesthesia for surgery and effects on postoperative pulmonary complications
LOS: Length of stay
NMBAs: Neuromuscular blocking agents
OLV: Open lung ventilation
PBW: Predicted body weight
PEEP: Positive end-expiratory pressure
Pplat: Plateau pressure
PPCs: Postoperative pulmonary complications
PROVEnet: Protective Ventilation Network
TLV: Total lung ventilation
TOF: Train–of–four stimulation
SpO_2_: Peripheral oxygen saturation
STROBE: Strengthening the reporting of observational studies in epidemiology
V_T_: Tidal volume

## References

[1] Weiser TG, Regenbogen SE, Thompson KD, Haynes AB, Lipsitz SR, Berry WR, Gawande AA (2008). An estimation of the global volume of surgery: a modelling strategy based on available data. Lancet.

[2] Bainbridge D, Martin J, Arango M, Cheng D, Evidence-based Peri-operative clinical outcomes research G (2012). Perioperative and anaesthetic-related mortality in developed and developing countries: a systematic review and meta-analysis. Lancet.

[3] Investigators LV (2017). Epidemiology, practice of ventilation and outcome for patients at increased risk of postoperative pulmonary complications: LAS VEGAS - an observational study in 29 countries. Eur J Anaesthesiol.

[4] Senturk M (2006). New concepts of the management of one-lung ventilation. Curr Opin Anaesthesiol.

[5] Severgnini P, Selmo G, Lanza C, Chiesa A, Frigerio A, Bacuzzi A, Dionigi G, Novario R, Gregoretti C, de Abreu MG (2013). Protective mechanical ventilation during general anesthesia for open abdominal surgery improves postoperative pulmonary function. Anesthesiology.

[6] Hemmes SN, Serpa Neto A, Schultz MJ (2013). Intraoperative ventilatory strategies to prevent postoperative pulmonary complications: a meta-analysis. Curr Opin Anaesthesiol.

[7] PROVE Network Investigators for the Clinical Trial Network of the European Society of Anaesthesiology; Sabrine N T Hemmes, Marcelo Gama de Abreu, Pelosi P, Schultz MJ. High versus low positive end-expiratory pressure during general anaesthesia for open abdominal surgery (PROVHILO trial): a multicentre randomised controlled trial. Lancet. 2014;384(9942):495–503. 10.1016/S0140-6736(14)60416-5 PMC6682759 24894577

[8] Young CC, Harris EM, Vacchiano C, Bodnar S, Bukowy B, Elliott RRD, Migliarese J, Ragains C, Trethewey B, Woodward A (2019). Lung-protective ventilation for the surgical patient: international expert panel-based consensus recommendations. Br J Anaesth.

[9] Hemmes SN, de Abreu MG, Pelosi P, Schultz MJ (2013). ESA clinical trials network 2012: LAS VEGAS--local assessment of Ventilatory management during general Anaesthesia for surgery and its effects on postoperative pulmonary complications: a prospective, observational, international, multicentre cohort study. Eur J Anaesthesiol.

[10] Arozullah AM, Daley J, Henderson WG, Khuri SF (2000). Multifactorial risk index for predicting postoperative respiratory failure in men after major noncardiac surgery. The National Veterans Administration Surgical Quality Improvement Program. Ann Surg.

[11] Arozullah AM, Khuri SF, Henderson WG, Daley J, Participants in the National Veterans Affairs Surgical Quality Improvement P (2001). Development and validation of a multifactorial risk index for predicting postoperative pneumonia after major noncardiac surgery. Ann Intern Med.

[12] Canet J, Gallart L, Gomar C, Paluzie G, Valles J, Castillo J, Sabate S, Mazo V, Briones Z, Sanchis J (2010). Prediction of postoperative pulmonary complications in a population-based surgical cohort. Anesthesiology.

[13] Smetana GW (2006). Preoperative pulmonary evaluation: identifying and reducing risks for pulmonary complications. Cleve Clin J Med.

[14] Guldner A, Kiss T, Serpa Neto A, Hemmes SN, Canet J, Spieth PM, Rocco PR, Schultz MJ, Pelosi P, Gama de Abreu M (2015). Intraoperative protective mechanical ventilation for prevention of postoperative pulmonary complications: a comprehensive review of the role of tidal volume, positive end-expiratory pressure, and lung recruitment maneuvers. Anesthesiology.

[15] Serpa Neto A, Hemmes SN, Barbas CS, Beiderlinden M, Biehl M, Binnekade JM, Canet J, Fernandez-Bustamante A, Futier E, Gajic O (2015). Protective versus conventional ventilation for surgery: a systematic review and individual patient data Meta-analysis. Anesthesiology.

[16] Futier E, Marret E, Jaber S (2014). Perioperative positive pressure ventilation: an integrated approach to improve pulmonary care. Anesthesiology.

[17] Slutsky AS, Ranieri VM (2013). Ventilator-induced lung injury. N Engl J Med.

[18] Kiss T, Wittenstein J, Becker C, Birr K, Cinnella G, Cohen E, El Tahan MR, Falcao LF, Gregoretti C, Granell M (2019). Protective ventilation with high versus low positive end-expiratory pressure during one-lung ventilation for thoracic surgery (PROTHOR): study protocol for a randomized controlled trial. Trials.

[19] Marret E, Cinotti R, Berard L, Piriou V, Jobard J, Barrucand B, Radu D, Jaber S, Bonnet F, the PPVsg (2018). Protective ventilation during anaesthesia reduces major postoperative complications after lung cancer surgery: a double-blind randomised controlled trial. Eur J Anaesthesiol.

[20] Lohser J, Slinger P (2015). Lung injury after one-lung ventilation: a review of the pathophysiologic mechanisms affecting the ventilated and the collapsed lung. Anesth Analg.

[21] von Elm E, Altman DG, Egger M, Pocock SJ, Gotzsche PC, Vandenbroucke JP, Initiative S (2014). The strengthening the reporting of observational studies in epidemiology (STROBE) statement: guidelines for reporting observational studies. Int J Surg.

[22] Bendixen HH, Hedley-Whyte J, Laver MB (1963). Impaired oxygenation in surgical patients during general anesthesia with controlled ventilation. A concept of atelectasis. N Engl J Med.

[23] Benumof J. Conventional and differential lung management of one-lung ventilation. In: Anesthesia for thoracic surgery. Philadelphia: Saunders, W.B; 1995. p. 799.

[24] Slinger P (2008). Perioperative lung injury. Best Pract Res Clin Anaesthesiol.

[25] Futier E, Constantin JM, Paugam-Burtz C, Pascal J, Eurin M, Neuschwander A, Marret E, Beaussier M, Gutton C, Lefrant JY (2013). A trial of intraoperative low-tidal-volume ventilation in abdominal surgery. N Engl J Med.

[26] Blank RS, Colquhoun DA, Durieux ME, Kozower BD, McMurry TL, Bender SP, Naik BI (2016). Management of one-lung Ventilation: impact of tidal volume on complications after thoracic surgery. Anesthesiology.

[27] Colquhoun DA, Naik BI, Durieux ME, Shanks AM, Kheterpal S, Bender SP, Blank RS, Investigators M (2018). Management of 1-lung ventilation-variation and trends in clinical practice: a report from the multicenter perioperative outcomes group. Anesth Analg.

[28] Blank RS, Lesh RE (2019). Low tidal volume ventilation in the surgical patient: not particularly low and perhaps not particularly protective. Anesth Analg.

[29] Ball L, Hemmes SNT, Serpa Neto A, Bluth T, Canet J, Hiesmayr M, Hollmann MW, Mills GH, Vidal Melo MF, Putensen C (2018). Intraoperative ventilation settings and their associations with postoperative pulmonary complications in obese patients. Br J Anaesth.

[30] Baudouin SV (2003). Lung injury after thoracotomy. Br J Anaesth.

[31] Cao C, Louie BE, Melfi F, Veronesi G, Razzak R, Romano G, Novellis P, Ranganath NK, Park BJ. Impact of pulmonary function on pulmonary complications after robotic-assisted thoracoscopic lobectomy. Eur J Cardiothorac Surg. 2020; 57(2):338–342. 10.1093/ejcts/ezz205 PMC7761531 31332434

[32] Im Y, Park HY, Shin S, Shin SH, Lee H, Ahn JH, Sohn I, Cho JH, Kim HK, Zo JI (2019). Prevalence of and risk factors for pulmonary complications after curative resection in otherwise healthy elderly patients with early stage lung cancer. Respir Res.

[33] Serpa Neto A, Hemmes SN, Barbas CS, Beiderlinden M, Fernandez-Bustamante A, Futier E, Hollmann MW, Jaber S, Kozian A, Licker M (2014). Incidence of mortality and morbidity related to postoperative lung injury in patients who have undergone abdominal or thoracic surgery: a systematic review and meta-analysis. Lancet Respir Med.

[34] Bluth T, Serpa Neto A, Schultz MJ, Pelosi P, Gama de Abreu M, Writing Committee for the PCGotPVNftCTNotESoA (2019). Effect of Intraoperative High Positive End-Expiratory Pressure (PEEP) With Recruitment Maneuvers vs Low PEEP on Postoperative Pulmonary Complications in Obese Patients: A Randomized Clinical Trial. JAMA.

[35] Pelosi P, Rocco PRM, Gama de Abreu M (2018). Close down the lungs and keep them resting to minimize ventilator-induced lung injury. Crit Care.

