# Supplementary material for: Intraoperative mechanical ventilation practice in thoracic surgery patients and its association with postoperative pulmonary complications: results of a multicenter prospective observational study
Source: BMC Anesthesiol. 2020 Jul 22;20:179. doi: 10.1186/s12871-020-01098-4 (PMC7373838; doi:10.1186/s12871-020-01098-4)
Supplement: Supplementary file 1 — Additional file 1: This PDF file contains a list of the LAS VEGAS Thorax study collaborators; Table S1. Participating centers; Table S2. ARISCAT Risk Score; Figure S1. Flowchart; Figure S2. Tidal volume, driving pressure, PEEP and FiO2 over time according to the use of one-lung ventilation or two-lung ventilation; Figure S3. Tidal volume, driving pressure, PEEP and FiO2 over time in endoscopic or open procedures; Figure S4. Tidal volume, driving pressure, PEEP and FiO2 over time according to the risk for PPC; Figure S5. Combinations of tidal volume and PEEP in the first three hours and last hour of surgery according to the use of one-lung ventilation or two-lung ventilation; Figure S6. Combinations of tidal volume and PEEP in the first three hours and last hour of surgery in endoscopic or non-endoscopic procedures; Figure S7. Combinations of tidal volume and PEEP in the first three hours and last hour of surgery according to the risk for PPC and Figure S8. Probability of hospital discharge according to development of PPC. [file 12871_2020_1098_MOESM1_ESM.docx]

**Intraoperative mechanical ventilation practice in thoracic surgery patients and its association with postoperative pulmonary complications: Results of a multicenter prospective observational study**

**ADDITIONAL FILE 1**

Christopher Uhlig, Ary Serpa Neto, Meta van der Woude, Thomas Kiss, Jakob Wittenstein, Benjamin Shelley, Helen Scholes, Michael Hiesmayer, Marcos Vidal Melo, Daniele Sances, Nesil Coskunfirat, Paolo Pelosi, Marcus Schultz, Marcelo Gama de Abreu; LAS VEGAS investigators; Protective Ventilation Network (PROVEnet); Clinical Trial Network of the European Society of Anaesthesiology

*Drs. Uhlig,Serpa Neto and van der Woude contributed equally to this manuscript.*

**Content**

List of LAS VEGAS Thorax study collaborators ……………………….………........pp. 2-5

Supplemental Table 1: Participating centers…………………..………………..…...pp. 6-8

Supplemental Table 2: ARISCAT Risk Score……...………………...…………….…..p. 9

Supplemental Figure 1: Flow chart……….…………….…………..………………..….p. 10

Supplemental Figure 2: Tidal volume, driving pressure, PEEP and FiO_2_ over time according to the use of one-lung ventilation or two-lung ventilation……….………...p. 11

Supplemental Figure 3: Tidal volume, driving pressure, PEEP and FiO_2_ over time in endoscopic or non-endoscopic procedures………………..…………….……………..p. 12

Supplemental Figure 4: Tidal volume, driving pressure, PEEP and FiO_2_ over time according to the risk for PPC…………………………….………………..…..………….p. 13

Supplemental Figure 5: Combinations of tidal volume and PEEP in the first three hours and last hour of surgery according to the use of one-lung ventilation or two-lung ventilation…………………………………………………………………………….……..p. 14

Supplemental Figure 6: Combinations of tidal volume and PEEP in the first three hours and last hour of surgery in endoscopic or non-endoscopic procedures……………………………………………………..…………………….……..p. 15

Supplemental Figure 7: Combinations of tidal volume and PEEP in the first three hours and last hour of surgery according to the risk for PPC….………………………….....p. 16

Supplemental Figure 8: Probability of hospital discharge according to development of PPC…………………..……………………………………………………………………..p. 17

**The LAS VEGAS Thorax Study Collaborators**

Austria

***Medical University Vienna*:** Michael Hiesmayr, Werner Schmid, Bernhard Urbanek

Belgium

***UCL - Cliniques Universitaires Saint Luc Brussels*:** David Kahn

***Universitary Hospital Brussels (UZ Brussel)*:** Jan Poelaert

***Ghent University Hospital, Gent*:** Stefan de Hert

***Maria Middelares, Gent*:** Stijn van de Velde

Croatia

***University Clinical Hospital Osijek, Osijek*:** Slavica Kvolik

***University Hospital Rijeka, Rijeka*:** Kazimir Juricic

***University Hospital, Medical school, “Sestre milosrdnice” (Sister of Charity), Zagreb*:** Branka Maldini

Czech Republic

***University Hospital Brno, Brno*:** Petr Stourac,

***University Hospital Hradec Kralove, Hradec Kralove*:** Jan Brujevic

***University Hospital Ostrava, Ostrava*:** Ivana Volfova

Egypt

***El Sahel Teaching hospital, Cairo*:** Samir el Kafrawy

Estonia

***North Estonia Medical Center, Tallinn*:** Eiko Herodes

***Tartu University Hospital, Tartu*:** Alar Sormus

France

***Saint Eloi University Hospital, Montpellier*:** Samir Jaber

Germany

***Fachkrankenhaus Coswig, Coswig*:** Jens Krassler

***University Hospital Carl Gustav Carus, Dresden*:** Marcelo Gama de Abreu, Christopher Uhlig, Thomas Kiss, Jakob Wittenstein, Gabrielle Müller, Jochen Schmitt

***Duesseldorf University Hospital, Heinrich-Heine University*:** Tanja Treschan

***Diakoniekrankenhaus Friederikenstift, Hannover*:** Andre Gottschalk

**University Hospital Bonn, Bonn:** Christian Putensen

**University Hospital Leipzig, Leipzig:** Hermann Wrigge

Greece

***General air force hospital, Athens*:** Georgios Antholopoulos

Italy

***Ospedale San. Paolo Bari, Bari*:** Caterina Valeria Pesce

***University of Bari “Aldo Moro”, Bari*:** Salvatore Grasso

***Azienda Ospedaliera per l’emergenza Cannizzaro, Catania*:** Luigi Giancarlo Vicari Sottosanti

***Azienda Ospedaliera – Universitaria Sant’Anna, Ferrara*:** Carlo Alberto Volta

***Ospedali Riuniti Di Foggia - University of Foggia, Foggia*:** Gilda Cinnella

***IRCCS AOU San Martino IST Hospital, University of Genoa, Genoa*:** Paolo Pelosi

***IRCCS San Raffaele Scientific Institute, Milano*:** Laura Pasin

***Istituto europeo di oncologia – ieo, Milano*:** Daniele Sances, Marco Venturino

***Ospedale Niguarda Ca'Granda Milano, Milano*:** Ines Arpino

***Ospedale San Paolo - University of Milano, Milano*:** Concezione Tommasino

***Policlinico "P. Giaccone", Palermo*:** Andrea Cortegiani

***Azienda Ospedaliero-Universitaria, Parma*:** Marco Baciarello

***Insubria University, Varese*:** Paolo Severgnini

Republic of Kosovo

***University Clinical Center of Kosova, Prishtina*:** Agreta Gecaj-Gashi

Lithuania

***Medical University Hospital, Hospital of Lithuanian University of Health Sciences, Kaunas*:** Aurika Karbonskiene

***Vilnius University Hospital - Institute of Oncology, Vilnius*:** Renatas Tikuisis

Netherlands

***Academic Medical Centre, University of Amsterdam*:** Sabrine N.T. Hemmes, Marcus Schultz, Markus W. Hollmann

***VU University Medical Center, Amsterdam*:** Christa Boer

***MC Haaglanden, Den Haag*:** Bas in  ’t  Veld

Norway

***Haukeland University Hospital, Bergen*:** Stefan Ivars

***Stavanger University Hospital, Stavanger*:** Amir Shafi-Kabiri

Panama

***Hospital Santo Tomás, Panama*:** Ruby Molina

Portugal

***Centro Hospitalar de Lisboa Central, E.P.E, Lisboa.*:** Maria de Lurdes Goncalves Castro

***Centro Hospitalar de Lisboa Ocidental, E.P.E. Hospital de S. Francisco Xavier, Lisboa*:** Suzana Parente

Romania

***Clinical Emergency Hospital of Bucharest, Bucharest*:** Ioana Marina Grintescu

***Fundeni Clinical institute - Anaesthesia and Intensive Care, Bucharest*:** Gabriela Droc

***Fundeni Clinical institute - Intensive Care Unit, Bucharest*:** Dana Tomescu

***University Emergency County Hospital Targu Mures, Targu Mures*:** Sanda-Maria Copotoiu

Russia

***Krasnoyarsk State Medical University, Krasnoyarsk*:** Alexey Gritsan

***Moscow Regional Research Clinical Institute, Moscow*:** Alexey Ovezov

***Reanimatology Research Institute n.a. Negovskij RAMS, Moscow*:** Valery Likhvantsev

Slovakia

***F.D. Roosevelt teaching Hospital, Banská Bystrica*:** Katarina Bruncakova

Slovenia

***University Medical Centre Ljubljana, Ljubljana*:** Jasmina Markovic-Bozic

Spain

***Hospital Sant Pau, Barcelona*:** Victoria Moral

***Hospital Universitari Germans Trias I Pujol, Barcelona*:** Jaume Canet

***University of Navarra, Pamplona*:** Pablo Monedero

***Corporacion Sanitaria Parc Tauli, Sabadell*:** Alberto Lisi

***Consorcio Hospital General Universitario de Valencia, Valencia*:** Manuel Granell

***Hospital Clinico Valencia, Valencia*:** Ernesto Pastor

***Hospital Universitario Rio Hortega, Valladolid*:** Cesar Aldecoa Alvares-Santullano

Sweden

**University Hospital Uppsula, Uppsala:** Göran Hedenstierna

Turkey

***Akdeniz University Hospital, Antalya*:** Nesil Coskunfirat, Neval Boztug

***Istanbul University, Istanbul medical faculty, Istanbul*:** Nuzhet Mert Senturk

***Dokuz Eylül Universitesi Tip Fakültesi, Izmir*:** Bahar Kuvaki

***Selcuk University faculty of medicine, Konya*:** Oguzhan Arun, Ali Saltali

Ukraine

***Institute Of Surgery And Transplantology, Kiev*:** Andriy Mazur

***Zaporizhzhia State Medical University, Zaporizhzhia*:** Sergiy Vorotyntsev

United Kingdom

***Golden Jubilee National Hospital, Clydebank, Scotland*:** Ben Shelley, Helen Scholes

***Royal Derby Hospital, Derby:*** David Rogerson

***The Princess Alexandra NHS Hospital Trust, Essex*:** Suresh Venkatesh

***Royal Devon and Exeter NHS Foundation Trust, Exeter*:** Matthew Rucklidge

***Royal Surrey County Hospital NHS Foundation Trust, Guildford*:** Maka Zuleika

***Kettering General Hospital NHS Foundation Trust, Kettering*:** Philip Watt

***Derriford Hospital Plymouth Hospitals NHS Trust, Plymouth*:** Danielle Franklin

***Royal Hallamshire Hospital, Sheffield*:** Gary Mills

***York Teaching Hospitals NHS Foundation Trust, York*:** Simon Davies

**Queen Mary University of London, London:** Rupert Pearse

United States

***University of Colorado School of Medicine/University of Colorado Hospital, Aurora*:** Ana Fernandez- Bustamante

***Massachusetts General Hospital, Boston*:** Marcos Vidal Melo, Luiz Fernando dos Reis Falcão

***Mayo Clinic, Rochester*:** Juraj Sprung

**Supplemental** **Table 1 Participating centers**

| **Country** | **Institution** | **City** |
| --- | --- | --- |
| Austria | Medical University, Vienna | Vienna |
| Belgium | UCL - Cliniques Universitaires Saint Luc Brussels | Brussels |
|  | Universitary Hospital Brussels (UZ Brussel) | Brussels |
|  | Ghent University Hospital | Gent |
|  | Maria Middelares Gent | Gent |
| Croatia | University Clinical Hospital Osijek | Osijek |
|  | University Hospital Rijeka | RIJEKA |
|  | University Hospital, Medical school, “Sestre milosrdnice” (Sister of Charity) | Zagreb |
| Czech Republic | Faculty Hospital Brno | Brno |
|  | University Hospital Ostrava | Ostrava |
|  | University Hospital Hradec Kralove | Hradec Kralove |
| Egypt | El Sahel Teaching hospital | Cairo |
| Estonia | Tartu University Hospital | Tartu |
|  | North Estonia Medical Center | Tallinn |
| France | Saint Eloi University Hospital, Montpellier | Montpellier |
| Germany | University Hospital Carl Gustav Carus | Dresden |
|  | Duesseldorf University Hospital | Duesseldorf |
|  | Diakoniekrankenhaus Friederikenstift | Hannover |
|  | Fachkrankenhaus Coswig Gmbh, centre for pneumology and thoracic surgery | Coswig |
| Greece | 251 General air force hospital | Athens |
| Italy | Ospedali Riuniti Di Foggia - University of Foggia | Foggia |
|  | Azienda ospedaliero – Universitaria Sant’Anna (Ferrara) | Ferrara |
|  | Insubria University | Varese |
|  | Policlinico "P Giaccone" (University of Palermo) | Palermo |
|  | Ospedale San Raffaele | Milano |
|  | University of Genoa | Genoa |
|  | Ospedale San. Paolo Bari | Bari |
|  | Azienda Ospedaliero-Universitaria | Parma |
|  | Istituto europeo di oncologia - ieo | Milano |
|  | University of Bari “Aldo Moro” | Bari |
|  | Azienda Ospedaliera per l’emergenza Cannizzaro | Catania |
|  | Ospedale Niguarda Ca'Granda Milano | Milano |
|  | Ospedale San Paolo - University of Milano | Milano |
| Lithuania | Vilnius University Hospital - Institute of Oncology | Vilnius |
|  | Kaunas Medical University Hospital, Hospital of Lithuanian University of Health Sciences | Kaunas |
| Netherlands | Academic Medical Centre, University of Amsterdam | Amsterdam |
|  | MCHaaglanden | Den Haag |
|  | VU University Medical Center | Amsterdam |
| Table continued on next page | | |
| **Supplemental** **Table 1 continued** | | |
| **Country** | **Institution** | **City** |
| Norway | Haukeland University Hospital | Bergen |
|  | Stavanger University Hospital | Stavanger |
| Panama | Hospital Santo Tomas | Panama |
| Portugal | Centro Hospitalar de Lisboa Central, EPE | Lisboa |
|  | [Centro Hospitalar de Lisboa Ocidental, E.P.E. Hospital de S. Francisco Xavier](http://www.hsfxavier.min-saude.pt/Homepage) | Lisboa |
| Republic of Kosovo | University Clinical Center of Kosova | Prishtina |
| Romania | Fundeni Clinical institute - Intensive Care Unit | Bucharest |
|  | Fundeni Clinical institute - Anesthesia and Intensive Care | Bucharest |
|  | Clinical Emergency Hospital of Bucharest | Bucharest |
|  | University Emergency County Hospital Targu Mures | Targu Mures |
| Russia | Reanimatology Research Institute n.a. Negovskij RAMS | Moscow |
|  | Krasnoyarsk State Medical University | Krasnoyarsk |
|  | Moscow Regional Research Clinical Institute | Moscow |
| Slovakia | F D Roosevelt Teaching Hospital | Banska Bystrica |
| Slovenia | University Medicine Centre Ljubljana | Ljubyana |
| Spain | University of Navarra | Pamplona |
|  | Consorcio Hospital General Universitario de Valencia | Valencia |
|  | Corporacion Sanitaria Parc Tauli | Sabadell |
|  | Hospital Santa Creu I Sant Pau | Barcelona |
|  | Hospital Clinico Valencia | Valencia |
|  | Hospital Universitari Germans Trias I Pujol | Barcelona |
|  | Hospital Universitario Rio Hortega | Valladolid |
| Turkey | Istanbul university, Istanbul medical faculty | Istanbul |
|  | Selcuk University faculty of medicine | Konya |
|  | Dokuz Eylül Universitesi Tip Fakültesi | Izmir |
|  | Akdeniz University Hospital | Antalya |
| Ukraine | Institute Of Surgery And Transplantology | Kiev |
|  | Zaporizhzhia State Medical University | Zaporizhzhia |
| United Kingdom | Derriford Hospita Plymouth Hospitals NHS Trust | Plymouth |
|  | Royal Devon and Exeter NHS Foundation Trust | Exeter |
|  | Golden Jubilee National Hospital | Clydebank, Scotland |
|  | Royal Derby Hospital | Derby |
|  | Royal Surrey County Hospital NHS Foundation Trust | Guiliford |
|  | Kettering General Hospital NHS Foundation Trust | Kettering |
|  | York Teaching Hospitals NHS Foundation Trust | York |
|  | The Princess Alexandra NHS Hospital Trust | ESSEX |
|  | Royal Hallamshire Hospital | Sheffield |
|  | Mayo Clinic | Rochester |
| Table continued on next page | | |
| **Supplemental Table 1 continued** | | |
| **Country** | **Institution** | **City** |
| United Kingdom | Massachusetts General Hospital, Boston, U.S.A. | Boston |
|  | University of Colorado School of Medicine/University of Colorado Hospital | Aurora, Colorado |

**Supplemental** **Table 2 ARISCAT Risk Score**

| **Independent predictors of risk for postoperative pulmonary complications identified in logistic regression model** | | | |
| --- | --- | --- | --- |
|  | **multivariate analysis** | **ß-coefficients** | **risk score**^†^ |
| Age (years) |  |  |  |
| ≤ 50 | 1 |  |  |
| 51 – 80 | 1.4 (0.6 - 3.3) | 0.331 | 3 |
| > 80 | 5.1 (1.9 - 13.3) | 1.619 | 16 |
| Preoperative SpO_2_ (%) |  |  |  |
| ≥ 96 | 1 |  |  |
| 91 – 95 | 2.2 (1.2 - 4.2) | 0.802 | 8 |
| ≤ 90 | 10.7 (4.1 - 28.1) | 2.375 | 24 |
| Respiratory infection in the last month | 5.5 (2.6 - 11.5) | 1.698 | 17 |
| Preoperative anaemia (≤ 100 g/L) | 3.0 (1.4 - 6.5) | 1.105 | 11 |
| Surgical incision |  |  |  |
| Peripheral | 1 |  |  |
| Upper abdominal | 4.4 (2.3 - 8.5) | 1.480 | 15 |
| Intrathoracic | 11.4 (4.9 - 26.0) | 2.431 | 24 |
| Duration of surgery (hours) |  |  |  |
| ≤ 2 | 1 |  |  |
| 2 to 3 | 4.9 (2.4 - 10.1) | 1.593 | 16 |
| > 3 | 9.7 (4.7 - 19.9) | 2.268 | 23 |
| Emergency procedure | 2.2 (1.04 - 4.5) | 0.768 | 8 |
| Modified according to Canet et al.^1^  ARISCATScore <26 low risk of PPCs, 26-44 moderate risk of PPCs, >44 high risk of PPCs  Abbreviations: ARISCAT: Assess Respiratory Risk in Surgical Patients in Catalonia risk score for PPCs, CI, confidence interval; OR, odds ratio; SpO_2_, oxyhaemoglobin saturation by pulse oximetry breathing air in supine position  ^†^ The simplified risk score was the sum of each logistic regression coefficient multiplied by 10, after rounding off its value | | | |
| 1. Canet J, Gallart L, Gomar C, et al. Prediction of postoperative pulmonary complications in a population-based surgical cohort. *Anesthesiology.* 2010;113(6):1338-1350 | | | |

**Supplemental** **Figure 1 Flow chart**

**
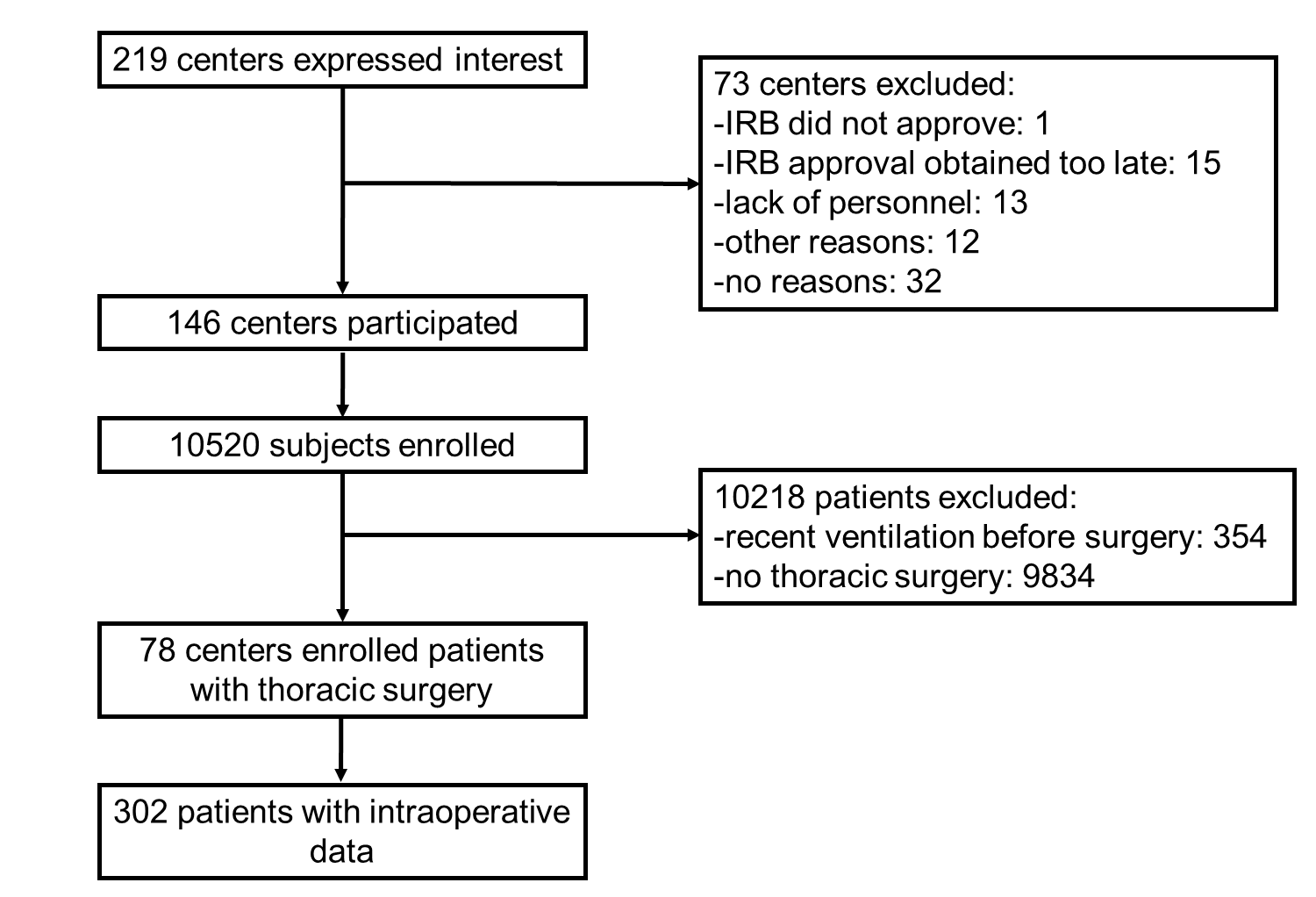
**

IRB: institutional review board

**Supplemental** **Figure 2 – Tidal volume, driving pressure, PEEP and FiO_2_ over time according to the use of one-lung ventilation or two-lung ventilation**

**
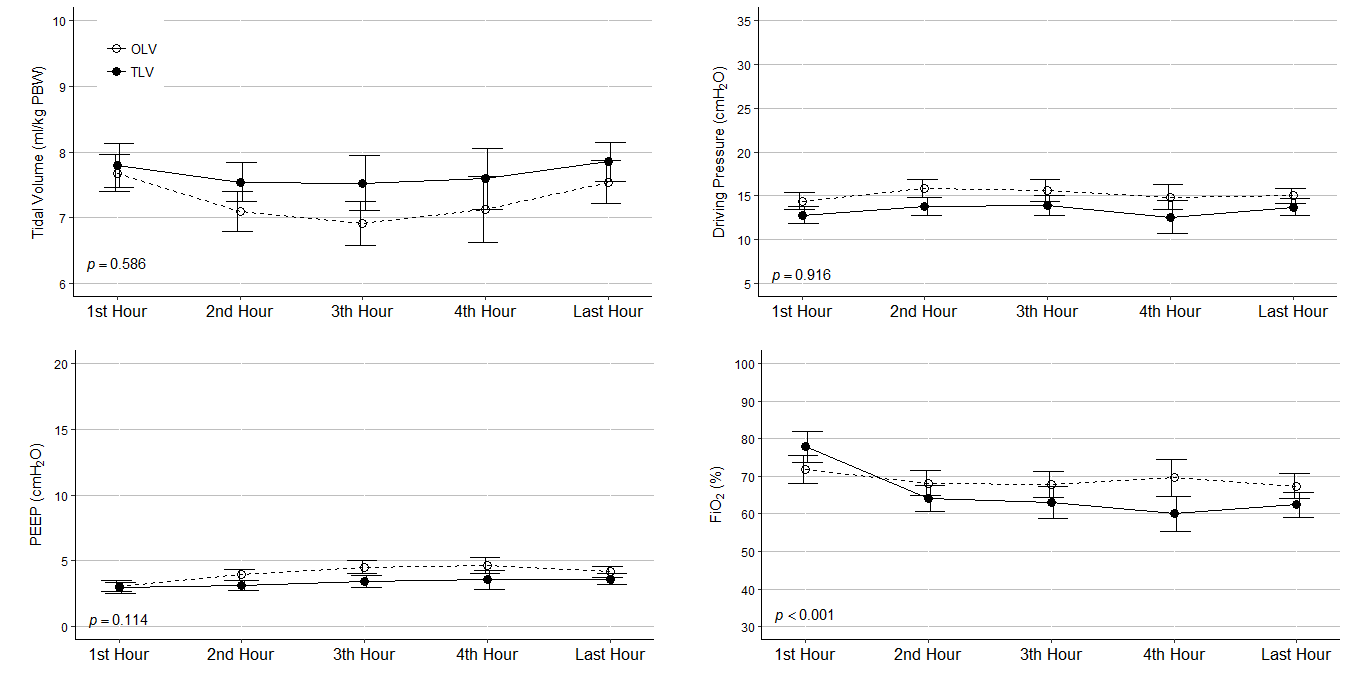
**

Mean and 95% confidence interval. *p* values from a mixed-effect linear model with participants as random-effect and group-time interaction as fixed effect. Driving pressure calculated as plato pressure – PEEP. PEEP: positive end-expiratory pressure; FiO_2_: inspired fraction of oxygen; OLV: one-lung ventilation; TLV: two-lung ventilation

**Supplemental Figure 3 – Tidal volume, driving pressure, PEEP and FiO_2_ over time in endoscopic or non-endoscopic procedures**

**
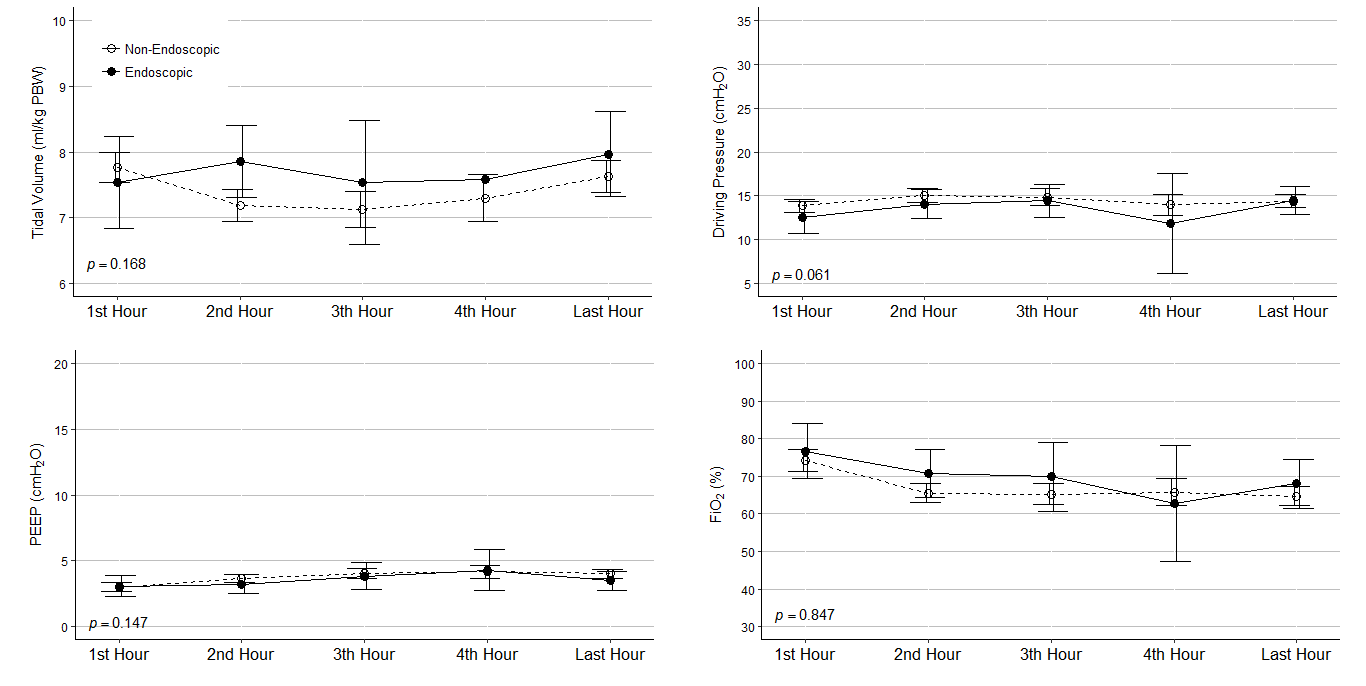
**

Mean and 95% confidence interval. *p* values from a mixed-effect linear model with participants as random-effect and group-time interaction as fixed effect. Driving pressure calculated as plateau pressure – PEEP. PBW: predicted body weight, PEEP: positive end-expiratory pressure; FiO_2_: inspired fraction of oxygen

**Supplemental Figure 4 – Tidal volume, driving pressure, PEEP and FiO_2_ over time according to the risk for PPC**

**
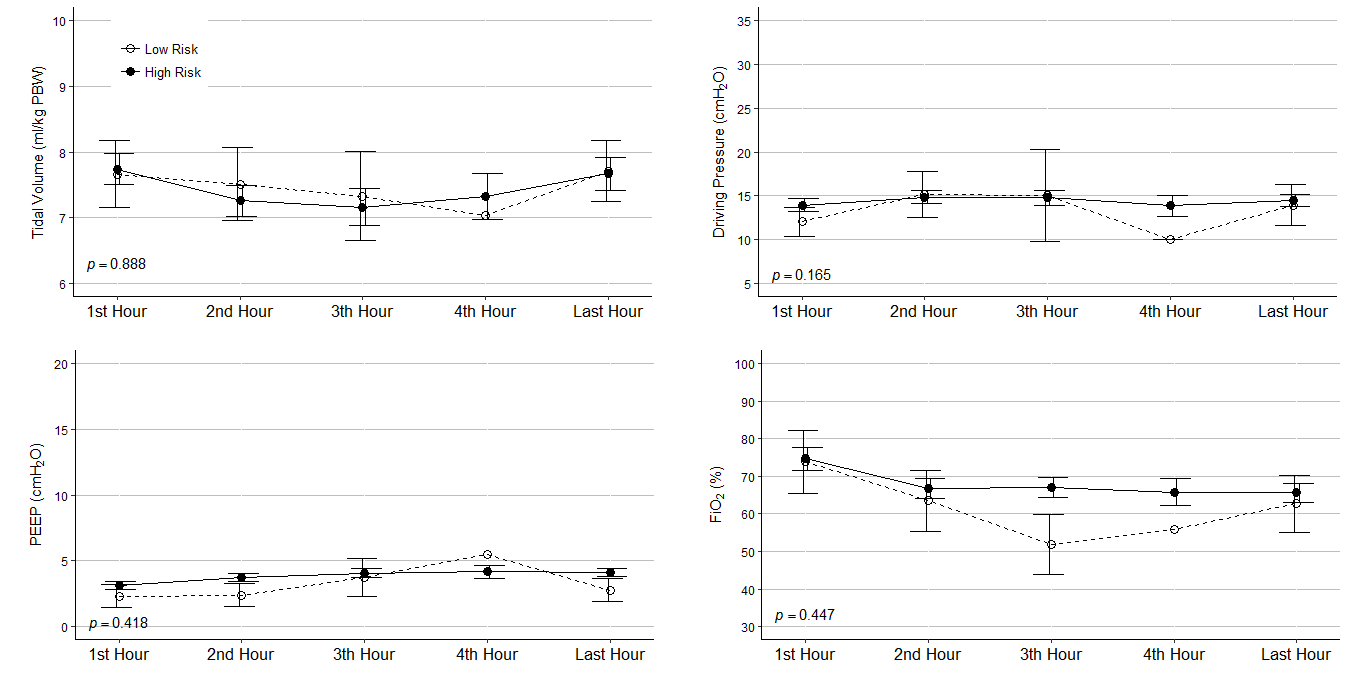
**Mean and 95% confidence interval. *p* values from a mixed-effect linear model with participants as random-effect and group-time interaction as fixed effect. Driving pressure calculated as plateau pressure – PEEP. Risk for PPC calculated from the ARISCAT score. PBW: predicted body weight; PEEP: positive end-expiratory pressure; FiO_2_: inspired fraction of oxygen; PPC: postoperative pulmonary complications

**Supplemental Figure 5 – Combinations of tidal volume and PEEP in the first three hours and last hour of surgery according to the use of one-lung ventilation or two-lung ventilation
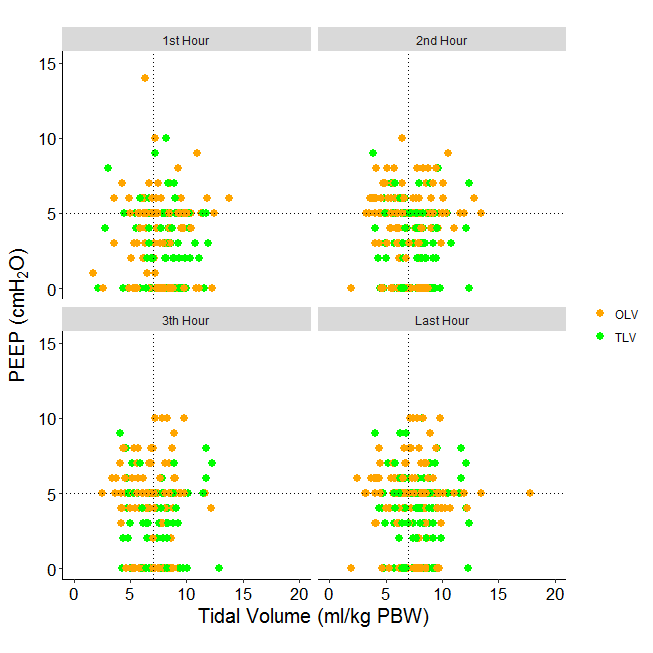
**

PBW: predicted body weight; PEEP: positive end-expiratory pressure; OLV: one-lung ventilation; TLV: two-lung ventilation

**Supplemental Figure 6 – Combinations of tidal volume and PEEP in the first three hours and last hour of surgery in endoscopic or non-endoscopic procedures**

**
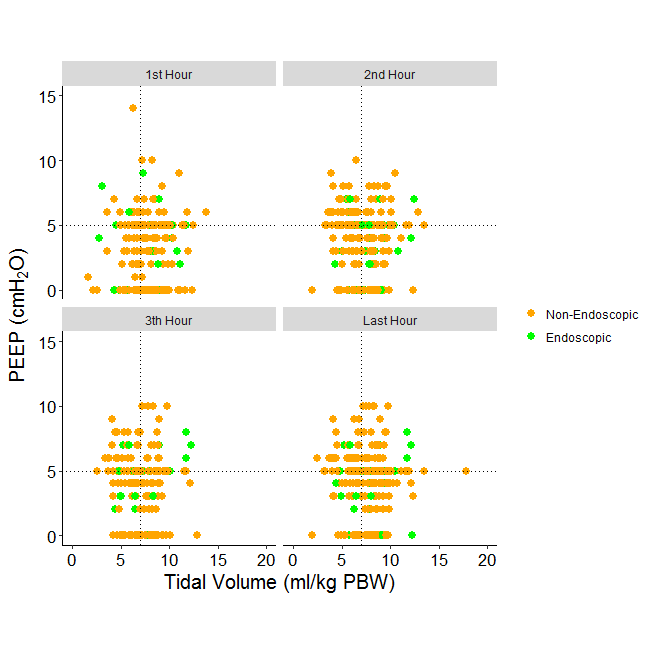
**

PBW: predicted body weight; PEEP: positive end-expiratory pressure

**Supplemental Figure 7 – Combinations of tidal volume and PEEP in the first three hours and last hour of surgery according to the risk for PPC**

**
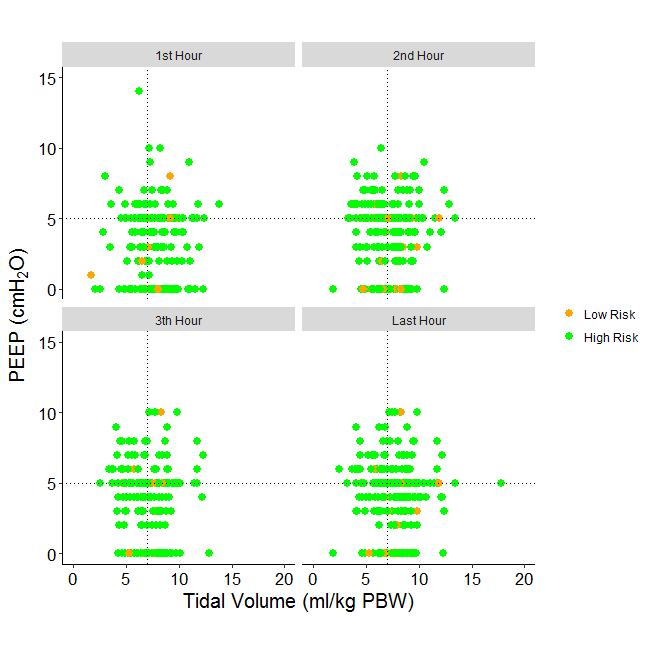
**

Risk for PPC calculated from the ARISCAT score. PBW: predicted body weight, PEEP: positive end-expiratory pressure; PPC: postoperative pulmonary complications

**Supplemental Figure 8 – Probability of hospital discharge according to development of PPC**

**
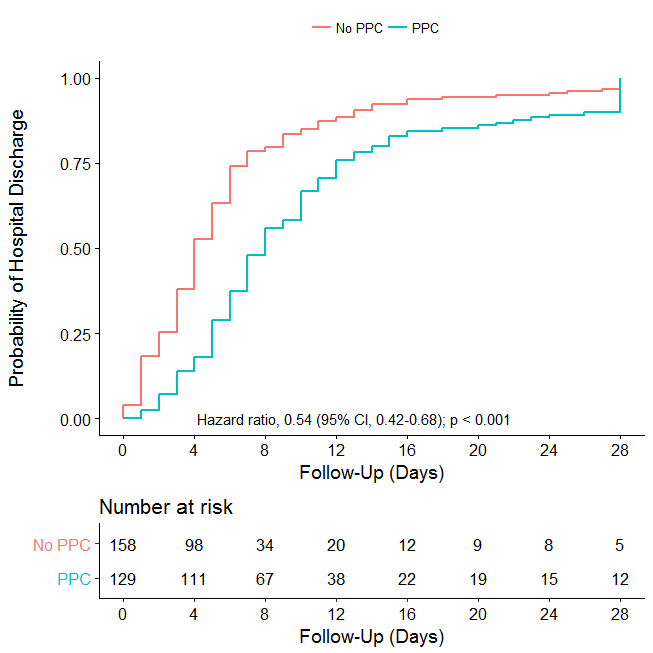
**

Non-adjusted hazard ratios. *PPC: postoperative pulmonary complications*
